# HIV-1 Vpr activates host CRL4-DCAF1 E3 ligase to degrade histone deacetylase SIRT7

**DOI:** 10.1186/s12985-021-01514-2

**Published:** 2021-03-01

**Authors:** Xiaohong Zhou, Christina Monnie, Maria DeLucia, Jinwoo Ahn

**Affiliations:** Department of Structural Biology and Pittsburgh Center for HIV Protein Interactions, University of Pittsburgh School of Medicine, Biomedical Science Tower 3, RM 1055, 3501 Fifth Ave., Pittsburgh, PA 15260 USA

**Keywords:** HIV-1, Vpr, Ubiquitin ligase, Protein degradation, Histone deacetylase, CRL4, SIRT7

## Abstract

**Background:**

Vpr is a virion-associated protein that is encoded by lentiviruses and serves to counteract intrinsic immunity factors that restrict infection. HIV-1 Vpr mediates proteasome-dependent degradation of several DNA repair/modification proteins. Mechanistically, Vpr directly recruits cellular targets onto DCAF1, a substrate receptor of Cullin 4 RING E3 ubiquitin ligase (CRL4) for poly-ubiquitination. Further, Vpr can mediate poly-ubiquitination of DCAF1-interacting proteins by the CRL4. Because Vpr-mediated degradation of its known targets can not explain the primary cell-cycle arrest phenotype that Vpr expression induces, we surveyed the literature for DNA-repair-associated proteins that interact with the CRL4-DCAF1. One such protein is SIRT7, a deacetylase of histone 3 that belongs to the Sirtuin family and regulates a wide range of cellular processes. We wondered whether Vpr can mediate degradation of SIRT7 via the CRL4-DCAF1.

**Methods:**

HEK293T cells were transfected with cocktails of plasmids expressing DCAF1, DDB1, SIRT7 and Vpr. Ectopic and endogeneous levels of SIRT7 were monitered by immunoblotting and protein–protein interactions were assessed by immunoprecipitation. For in vitro reconstitution assays, recombinant CRL4-DCAF1-Vpr complexes and SIRT7 were prepared and poly-ubiqutination of SIRT7 was monitored with immunoblotting.

**Results:**

We demonstrate SIRT7 polyubiquitination and degradation upon Vpr expression. Specifically, SIRT7 is shown to interact with the CRL4-DCAF1 complex, and expression of Vpr in HEK293T cells results in SIRT7 degradation, which is partially rescued by CRL inhibitor MNL4924 and proteasome inhibitor MG132. Further, in vitro reconstitution assays show that Vpr induces poly-ubiquitination of SIRT7 by the CRL4-DCAF1. Importantly, we find that Vpr from several different HIV-1 strains, but not HIV-2 strains, mediates SIRT7 poly-ubiquitination in the reconstitution assay and degradation in cells. Finally, we show that SIRT7 degradation by Vpr is independent of the known, distinctive phenotype of Vpr-induced cell cycle arrest at the G2 phase,

**Conclusions:**

Targeting histone deacetylase SIRT7 for degradation is a conserved feature of HIV-1 Vpr. Altogether**,** our findings reveal that HIV-1 Vpr mediates down-regulation of SIRT7 by a mechanism that does not involve novel target recruitment to the CRL4-DCAF1 but instead involves regulation of the E3 ligase activity.

## Background

HIV-1 encodes several accessory proteins that counteract anti-viral intrinsic immunity [1, 2]. Among these accessory proteins, Vpr is enigmatic, and the precise mechanisms by which it antagonizes antiviral responses are not clearly understood. Currently, it is widely accepted that Vpr directly loads post-replication DNA repair proteins onto DCAF1, a substrate receptor subunit of Cullin 4 RING E3 ubiquitin ligase (CRL4), thereby mediating their proteasome-dependent degradation [3, 4]. Further, Vpr may also activate the CRL4 such that cellular factors interacting with DCAF1 during homeostasis are degraded upon HIV infection [5–7]. However, the breadth of biological phenotypes associated with Vpr are not fully explained by degradation of known cellular targets, suggesting that additional substrates of the Vpr-bound CRL4-DCAF1 remain to be identified.

Sirtuin 7 (SIRT7) is a member of the Sir2 protein family, possessing a NAD^+^-dependent protein deacetylase catalytic core domain [8, 9]. Localized in the nucleus, SIRT7 deacetylates several nuclear proteins including histone 3 at lysine 18, which facilitates recruitment of DNA repair proteins at damaged sites [10]. Considering that SIRT7 was previously shown to inhibit proteasome-dependent degradation of nuclear receptor TR4 by direct interaction with the CRL4-DCAF1 [11] and possesses activities to regulate transcription, DNA damage repair, and DNA replication [12–14], we explored the possibility that SIRT7 is a target of Vpr. In the present study, we establish a biochemical link between SIRT7 and CRL4-DCAF1-Vpr. Specifically, using in vitro reconstitution assays, we show poly-ubiquitination of SIRT7 by the Vpr-bound CRL4-DCAF1 and Vpr-mediated degradation of SIRT7 in a CRL4- and proteasome-dependent manner. Importantly, Vpr isolated from various HIV-1 strains and their ancestral virus, SIVcpz (Simian Immunodeficieny Virus infecting chimpanzee), but not from HIV-2, induces degradation of SIRT7, suggesting that Vpr-dependent degradation of SIRT7 has been conserved during zoonotic events.

## Methods

### Cloning and plasmid constructions

HIV-1 NL4-3 Vpr and its mutants, various Vpr clones from HIV-1 YU2, LAI, Q23, M, N and O group, SIVcpz PTT, and PTS were cloned into the pcDNA3.1 vector (Thermo Fisher Scientific) with an HA epitope tag at the N-terminus. HA-tagged SIRT7, FLAG and HA-tagged SIRT7, FLAG or HA-tagged DCAF1, and Myc and HA-tagged DDB1 were cloned into the pcDNA3.1 vector. For *E.coli* expression, SIRT7 with 6X His tag at the N-terminus was cloned into the pET28 (EMD Bioscience). All other clones were described previously [5, 15, 16].

### Protein purification

SIRT7 in the pET28 vector was expressed in *E. coli* Rosetta 2 (DE3) in autoidunction medium at 18 °C for 16 h. SIRT7 and all other proteins were purified as described previously [5, 15, 16].

### In vitro ubiquitination assays

E1 (UBA1, 0.5 µM), E2 (Ubc-5Hb, 5 µM), and appropriate E3 ubiquitin ligase complexes (mixtures of CUL4A-RBX1 and DDB1-DCAF1, DDB1-DCAF1-Vpr or DDB1-DCAF1-Vpx at each 0.5 µM) were incubated with 1 µM of SIRT7 and 5 µM of ubiquitin in a buffer containing 10 mM Tris–HCl, pH 7.5, 150 mM NaCl, 5% glycerol, 20 units/mL pyrophosphatase, 1 mM TCEP, and 5 mM ATP. The reaction mixtures were terminated with SDS-PAGE sample loading buffer after 5 15, and 45 min. The degree of ubiquitination was detected by immunoblotting with the anti-SIRT7 antibody.

### Mammalian cell lines, transfection, immunoprecipitation and western blotting

Human embryonic kidney cell lines (HEK293T from ATCC) were maintained in advanced DMEM, supplemented with Glutamine and 10% (v/v) fetal bovine serum. A day before transfection, approximately 3 × 10^6^ cells were seeded on a 100 mm dish. Transfection was performed with a mixture of pcDNA plasmids expressing specific proteins, as indicated, using Lipofectamine 3000 (Thermo Fisher Scientific) with empty pcDNA vector plasmid as a balance. After 48 h, cells were harvested and lysed with sonication in the lysis buffer (50 mM Tris, 150 mM NaCl, 0.5% NP-40, pH 7.5) with complete protease inhibitor mixture (Sigma). Some cell lysate was mixed with loading buffer and heated at 95 °C for 5 min. For immunoprecipitation, the cleared cell lysate was incubated with 20 µL of anti-FLAG magnetic beads (Sigma) with shaking at 4 °C for 5 h. The beads were washed with the lysis buffer three times and bound proteins were eluted with FLAG peptides at 0.1 mg/mL. Samples were subjected to SDS-PAGE and Western Blotting analysis with appropriate antibodies. Antibodies used in the currernt study included anti-HA (COVANCE), anti-FLAG (Abnova), anti-DDB1 (Sigma), anti-Actin (Sigma), anti-DCAF1 (Santa Cruz), anti-SIRT7 (Santa Cruz), anti-Goat IgG (Santa Cruz), anti-Rabbit IgG (Sigma), and anti-Mouse IgG (Sigma).

### Cell cycle assays

HEK293T cells seeded on 6-well plates were transfected with pcDNA plasmid expressing Vpr, Vpx or empty vector. Cells were collected 48 h after transfection. After fixation with 70% ethanol for 30 min and two washes with PBS, cells were resuspended in 0.5 ml 10 μg/ml propidium iodide staining solution. After staining for 30 min at room temperature, cell profiles were analyzed by flow cytometry (BD LSRII SORP). Cell cycle data were analyzed with FlowJo software (Tree Star Inc., Ashland, OR).

## Results

### SIRT7 is a CRL4-DCAF1 binding protein

**Fig. 1:**
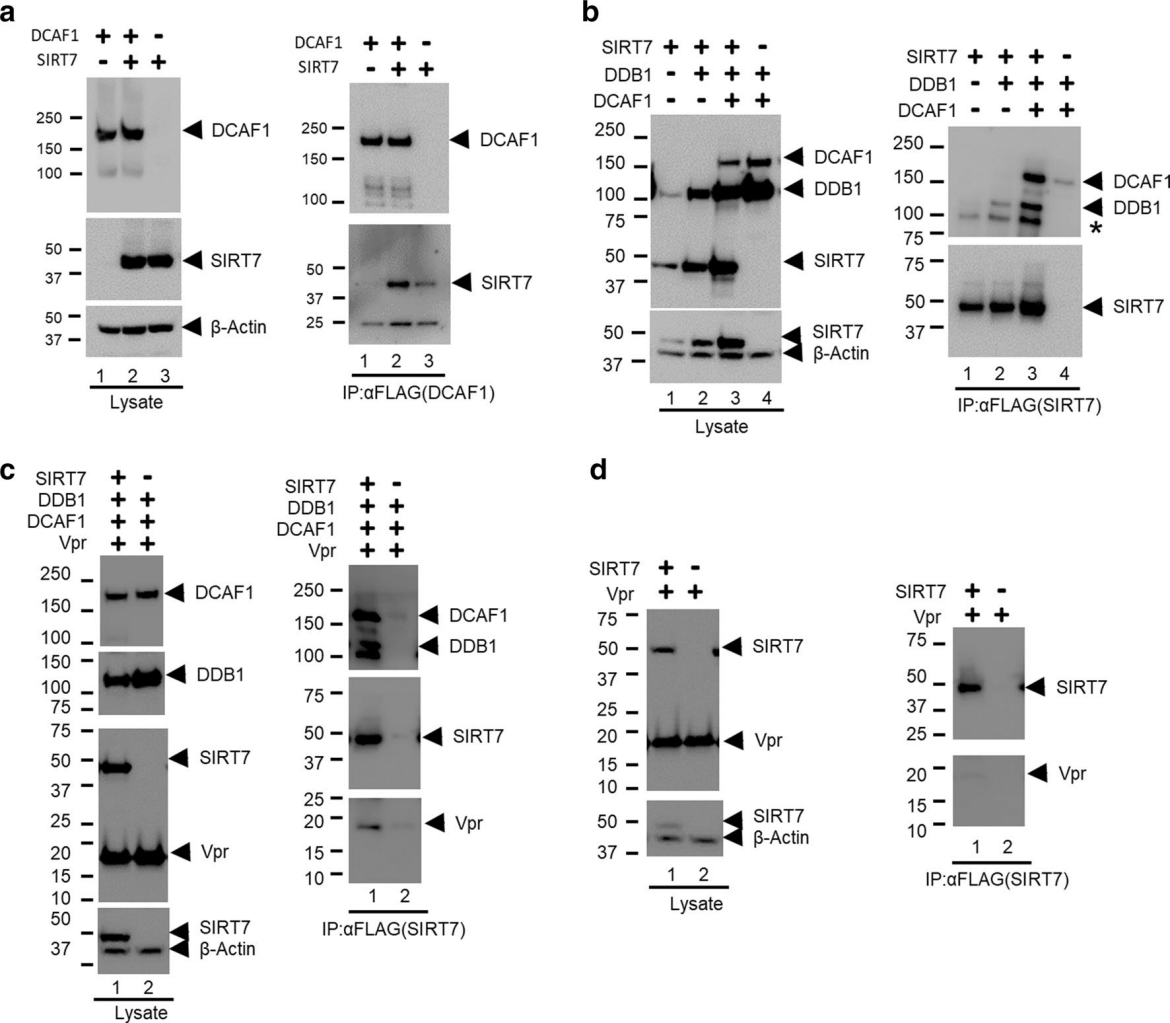
SIRT7 binds to the substrate receptor/adaptor complex, DCAF1/DDB1 of CRL4. **a** Control plasmids or plasmids expressing DCAF1 or SIRT7 were transiently co-transfected into HEK293T cells as indicated. 48 h later, cells were harvested and cell lysates were prepared for immunoprecipitation (IP) with anti-FLAG antibody. Both lysate and IP elution fractions were subjected to SDS-PAGE and immunoblotting with appropriate antibodies. **b** Control plasmids or plasmids expressing SIRT7, DCAF1 or DDB1 were transiently co-transfected into HEK293T cells as indicated. Cells were treated as in **a**. Endogenous DDB1 is indicated (*). **c** The mixture of plasmids expressing DDB1, DCAF1, and Vpr were co-transfected into HEK293T cells with or without plasmids expressing SIRT7. Immunoprecipitation of SIRT7 was performed with anti-FLAG antibody. **d** Plasmids expressing Vpr were co-transfected with plasmids expressing SIRT7 or control plasmids into HEK293T cells. SIRT7 proteins were immunoprecipitated with anti-FLAG antibody and probed with immunoblotting. Experiments were repeated with two to three times with similar results

SIRT7 was previously reported to interact with the CRL4-DCAF1 and modulate its ubiquitin ligase activity [11, 17, 18]. Consistent with those findings, in co-immunoprecipitation experiments, we observed an interaction between DCAF1 and SIRT7, despite some non-specific interaction of SIRT7 and anti-FLAG antibody beads (Fig. 1a). Further, co-immunoprecipitation experiments against SIRT7 after co-expression of DDB1 or DDB1 and DCAF1 suggest that SIRT7 is a binding partner of the CRL4-DCAF1 (Fig. 1b).

We next wondered whether SIRT7 would still interact with the CRL4-DCAF1 in the presence of Vpr. Co-immunoprecipitation experiments showed that SIRT7 indeed forms a stable complex with DDB1, DCAF1 and Vpr (Fig. 1c). However, we did not see appreciable interaction between Vpr and SIRT7 (Fig. 1d). Together, these data suggest that both SIRT7 and Vpr form a stable complex with the CRL4-DCAF1.

### SIRT7 is poly-ubiquitinated by the CRL4-DCAF1 in a Vpr-dependent manner for proteasome-dependent degradation

**Fig. 2:**
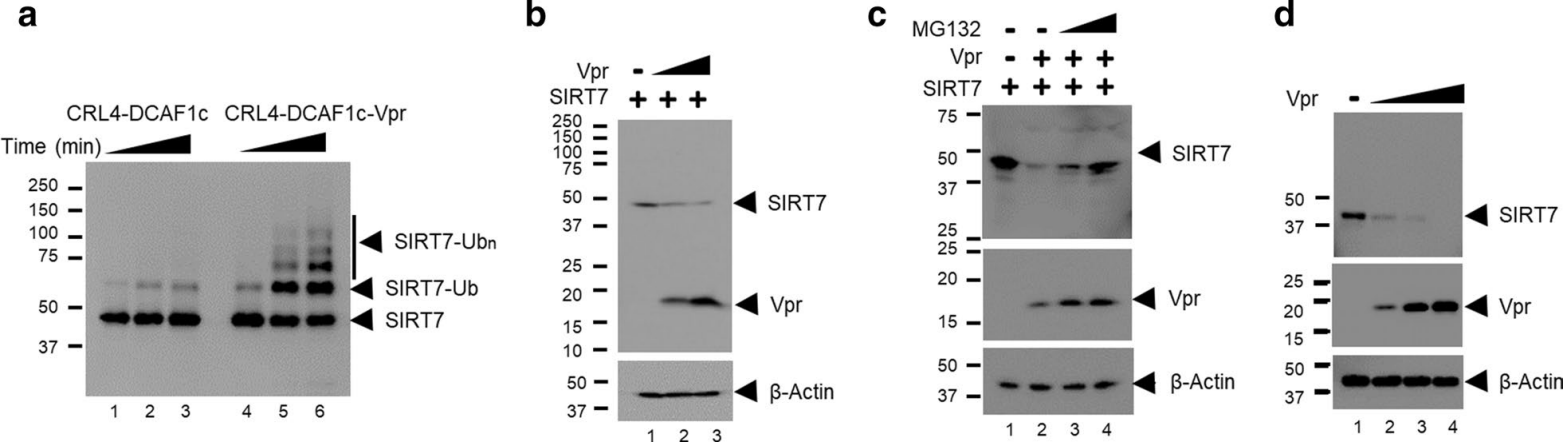
Vpr promotes poly-ubiquitination and degradation of SIRT7. **a** In vitro ubiquitination assays of SIRT7 with CRL4-DCAF1c (DCAF1 residue 1045–1396) or CRL4-DCAF1c-Vpr. Poly-ubiquitinated SIRT7 species are indicated (SIRT7-Ubn). **b** A constant amount of plasmids expressing SIRT7 were transfected into HEK293T cells with increasing amounts of Vpr-expressing plasmids. Cells were harvested after 48 h, and cell lysate was prepared and subjected to SDS-PAGE and immunoblotting. **c** Control plasmids or plasmids expressing SIRT7 or Vpr were co-transfected into HEK293T cells. 40 h later, cells were treated with 10 μM MG132. Cells were harvested after an additional 8 h, and cell lysate was prepared for SDS-PAGE and immunoblotting. **d** Plasmids expressing Vpr were transfected into HEK293T cells. All experiments were repeated two to three times with equivalent results

Vpr can mediate proteasome-dependent degradation of several cellular targets by two distinctive mechanisms: Vpr, associated with DCAF1, directly interacts and recruits cellular targets for poly-ubiquitination by the CRL4, or Vpr, by binding DCAF1, activates the E3 ligase activity and mediates poly-ubiquitination of DCAF1-interacting proteins (see Fig. 6 and Discussion). Since we found that both SIRT7 and Vpr simultaneously interact with DDB1 and DCAF1 (Fig. 1c), we asked whether Vpr modulates the ubiquitin ligase activity of CRL4-DCAF1 for poly-ubiquitination of SIRT7. To that end, we performed in vitro ubiquitination assays with reconstituted CRL4-DCAF1 and CRL4-DCAF1-Vpr complexes (Fig. 2a). Here, we used the C-terminal domain of DCAF1 (residues 1045–1396), which is sufficient for interacting with DDB1, Vpr and SIRT7. Poly-ubiquitination of SIRT7 was apparent only in the presence of Vpr (Compare lane 1–3 versus lanes 4–6 in Fig. 2a). Transient expression of SIRT7 and Vpr resulted in down-regulation of SIRT7 in a Vpr-dose dependent manner (Fig. 2b). Treatment of those cells with proteasome inhibitor, MG132 moderated Vpr-dependent down-regulation of SIRT7 (Fig. 2c). Further, the level of endogenous SIRT7 protein decreased with increasing ectopic expression of Vpr (Fig. 2d). Taken together, these data suggest that Vpr enhances the activity of CRL4-DCAF1 to poly-ubiquitinate SIRT7 for proteasome-dependent degradation.

**Fig. 3:**
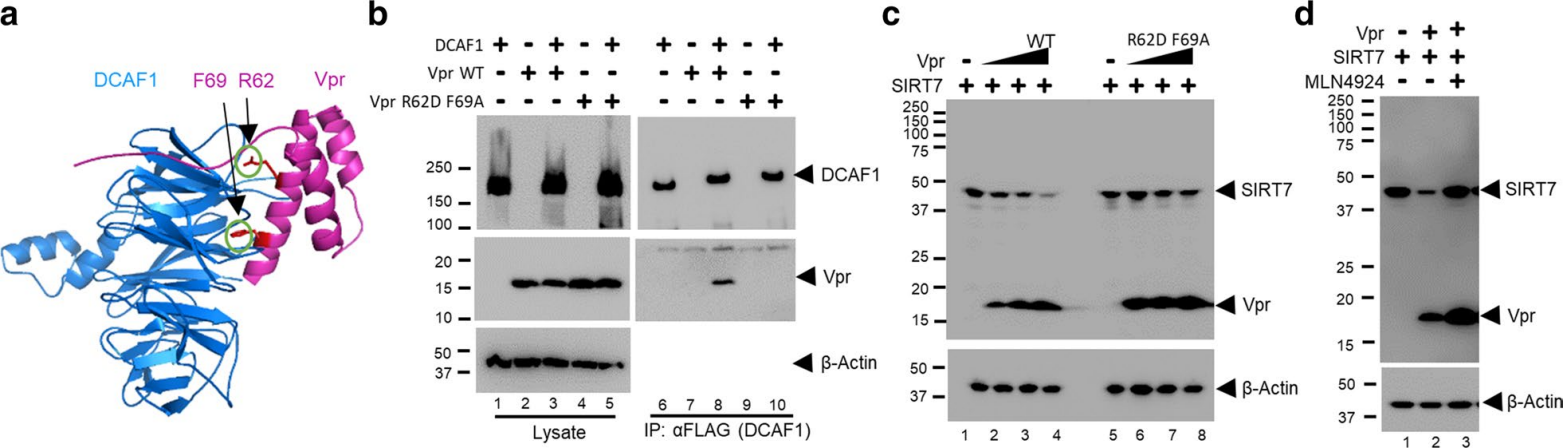
Vpr-mediated SIRT7 degradation is DCAF1- and CRL4-dependent. **a** DCAF1 (blue) and Vpr (magenta) binding interface. The complex structure of DCAF1 and Vpr was extracted from the crystal structure of DDB1/DCAF1/Vpr/UNG2 (PDB: 5JK7). DCAF1-interacting residues of Vpr, F69 and R62 are shown in stick representation. **b** Plasmids expressing DCAF1 were co-transfected into HEK293T with plasmids expressing wild-type (WT) or R62D/F69A mutant Vpr. Cell lysates were subjected to immunoprecipitation with anti-FLAG antibody. **c** Plasmids expressing SIRT7 were co-transfected with control plasmids or plasmids expressing WT or R62D/F69A mutant Vpr at escalating concentrations. Cell lysate was analyzed by immunoblotting. **d** Plasmids expressing SIRT7 or Vpr were co-transfected into HEK293T cells. 40 h later, cells were treated with 1 μM MLN4924 and incubated for an additional 8 h. Cell lysate was analyzed by immunoblotting. All experiments were repeated two to three times with similar results

To further understand the role of Vpr in mediating SIRT7 down-regulation, we generated a Vpr mutant R62D/F69A. These two residues of Vpr reside at the DCAF1 binding interface (Fig. 3a). We previously showed that single R62D or F69A mutation affected Vpr interaction with DCAF1, respectively, and F69A mutation reduced Vpr-mediated Exo1 association with DDB1 and DCAF1 [15, 27]. The Vpr mutant does not form a stable complex with DCAF1 (Fig. 3b) and cannot mediate down-regulation of SIRT7 (Fig. 3c). Further, treatment of cells ectopically expressing Vpr and SIRT7 with the CRL inhibitor, MLN4924 resulted in elevated SIRT7 level (Fig. 3d). Altogether, these data suggest that Vpr binding to DCAF1 is necessary to mediate SIRT7 degradation in a CRL4-dependent manner.

### SIRT7 degradation is a conserved feature of HIV-1 Vpr and SIVcpz Vpr

**Fig. 4:**
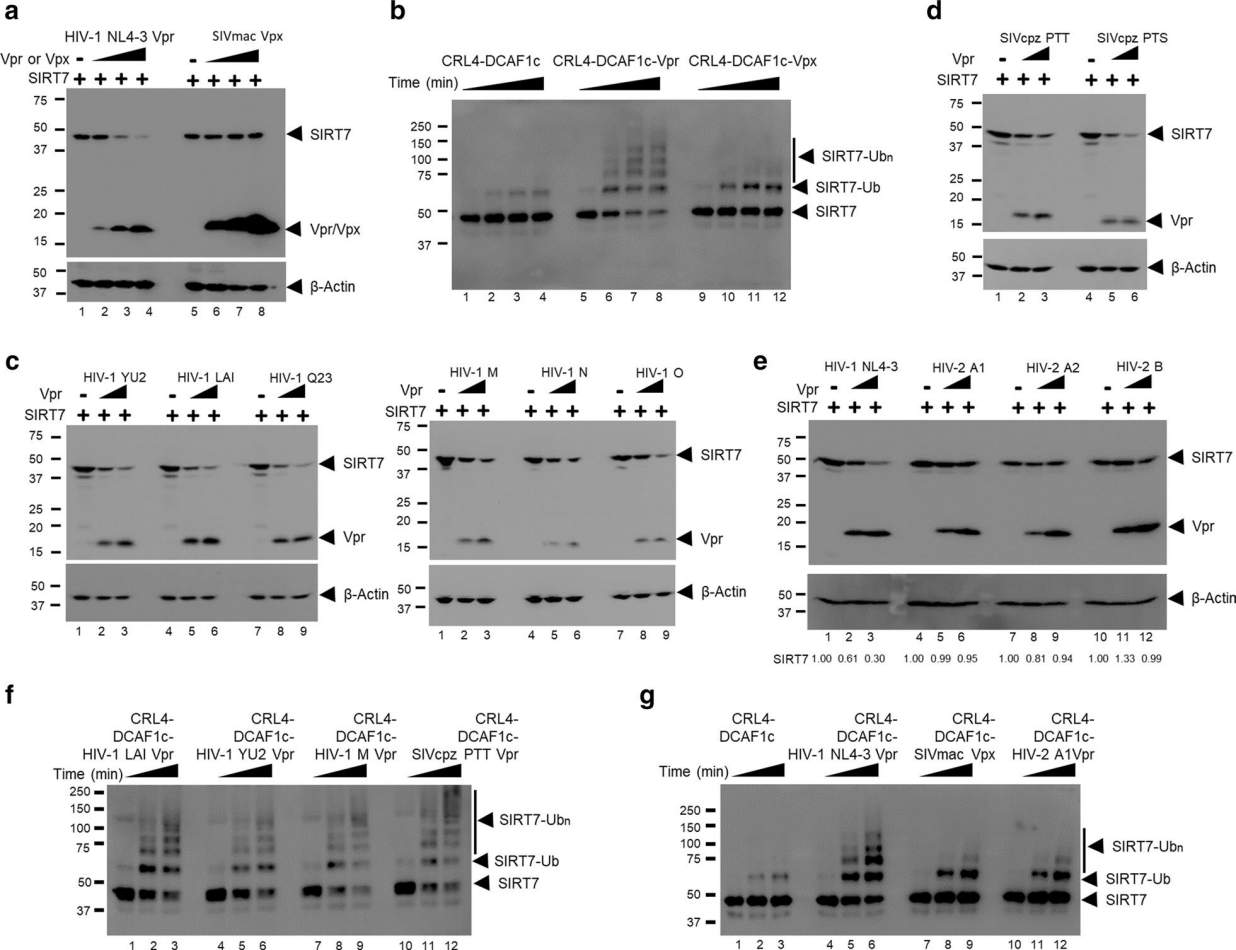
SIRT7 degradation is a conserved feature of HIV-1 Vpr. **a** Plasmids expressing SIRT7 were co-transfected with increasing amounts of plasmids expressing HIV-1 NL4-3 Vpr or SIVmac Vpx. Cell lysate was prepared and subjected to SDS-PAGE and immunoblotting. **b** In vitro ubiquitination assays of SIRT7 with CRL4-DCAF1c, CRL4-DCAF1c-Vpr (HIV-1 NL4-3), or CRL4-DCAF1c-Vpx (SIVmac). Poly-ubiquitinated SIRT7 species are indicated (SIRT7-Ubn). **c–e** Plasmids expressing SIRT7 were co-transfected into HEK293T cells with increasing amounts of plasmids expressing HIV-1 YU2, LAI, Q23, M, N, or O Vpr (**c**), SIVcpz PTT or PTS Vpr (**d**), or HIV-1 NL4-3, HIV-2 A1, HIV-2 A2, or HIV-2 B Vpr (**e**). Relative intensity of bands corresponding to SIRT7 was quantified (**e**). **f**, In vitro ubiquitination assays of SIRT7 with CRL4-DCAF1c in complex with HIV-1 LAI, YU2, M, or SIVcpz PTT Vpr. **g** In vitro ubiquitination assays of SIRT7 with CRL4-DCAF1c or CRL4-DCAF1c in complex with HIV-1 NL4-3 Vpr, SIVmac Vpx or HIV-2 A1 Vpr. All experiments were repeated two to three times with similar results

The CRL4-DCAF1 is also usurped by an ortholog of HIV-1 Vpr, HIV-2/SIVmac (SIV isolated from macaque monkey) Vpx, which induces degradation of SAMHD1 and HUSH complex [19–22]. Structural studies suggested that both Vpr and Vpx essentially bind DCAF1 in the same manner, using a common interface, and recruit their respective cellular targets using unique interfaces [15, 23–25]. Thus, we asked whether Vpx can also induce degradation SIRT7 by activating the CRL4-DCAF1. Surprisingly, SIVmac Vpx, even at a higher level than Vpr, did not enhance degradation of SIRT7 (Fig. 4a). In vitro ubiquitination confirmed that Vpx does not significantly enhance the CRL4-DCAF1 activity to mediate poly-ubiquitination of SIRT7 (Fig. 4b). Thus, the data suggest that CRL4-DCAF1 activation is a unique function inherent only to HIV-1 Vpr.

All the above studies were performed with Vpr isolated from the HIV-1 NL4-3 strain. To explore whether Vpr-dependent SIRT7 degradation is evolutionarily conserved among various HIV-1 strains, we co-expressed SIRT7 with Vpr isolated from HIV-1 YU2, LAI, Q23, M, N or O group (Fig. 4c). Each tested Vpr protein reduced the SIRT7 level in a dose-dependent manner. Further, Vpr isolated from SIVcpz PTT and PTS strains, evolutionary predecessors of HIV-1, also reduced the level of SIRT7 (Fig. 4d). However, Vpr isolated from HIV-2 A1, A2 or B strain, that are closely related SIV isolated from sooty mangabeys, did not efficiently reduce SIRT7 level (Fig. 4e). In line with our observation, recent quantitative proteomics with CEM-T4 T cells transduced with VSVg-pseudotyped HIV containing HIV-1/SIVcpz Vpr, but not other SIV or HIV-2 Vpr, showed reduction in SIRT7 cellular level, [26]. In vitro ubiquitination assays of SIRT7 were performed with CRL4-DCAF1 in complex with various Vpr proteins and confirmed that SIRT7 is targeted for poly-ubiquitination (Fig. 4f). On the other hand, Vpx isolated from SIVmac and Vpr isolated from HIV-2 A1 did not siginificantly enhance the ubiquitin ligase activity of CRL4-DCAF1 (Fig. 4g). Thus, taken altogether, the data suggest that SIRT7 degradation is a conserved function of Vpr in HIV-1 and SIVcpz.

### SIRT7 degradation and G2 arrest are independent functions of HIV-1 Vpr

**Fig. 5:**
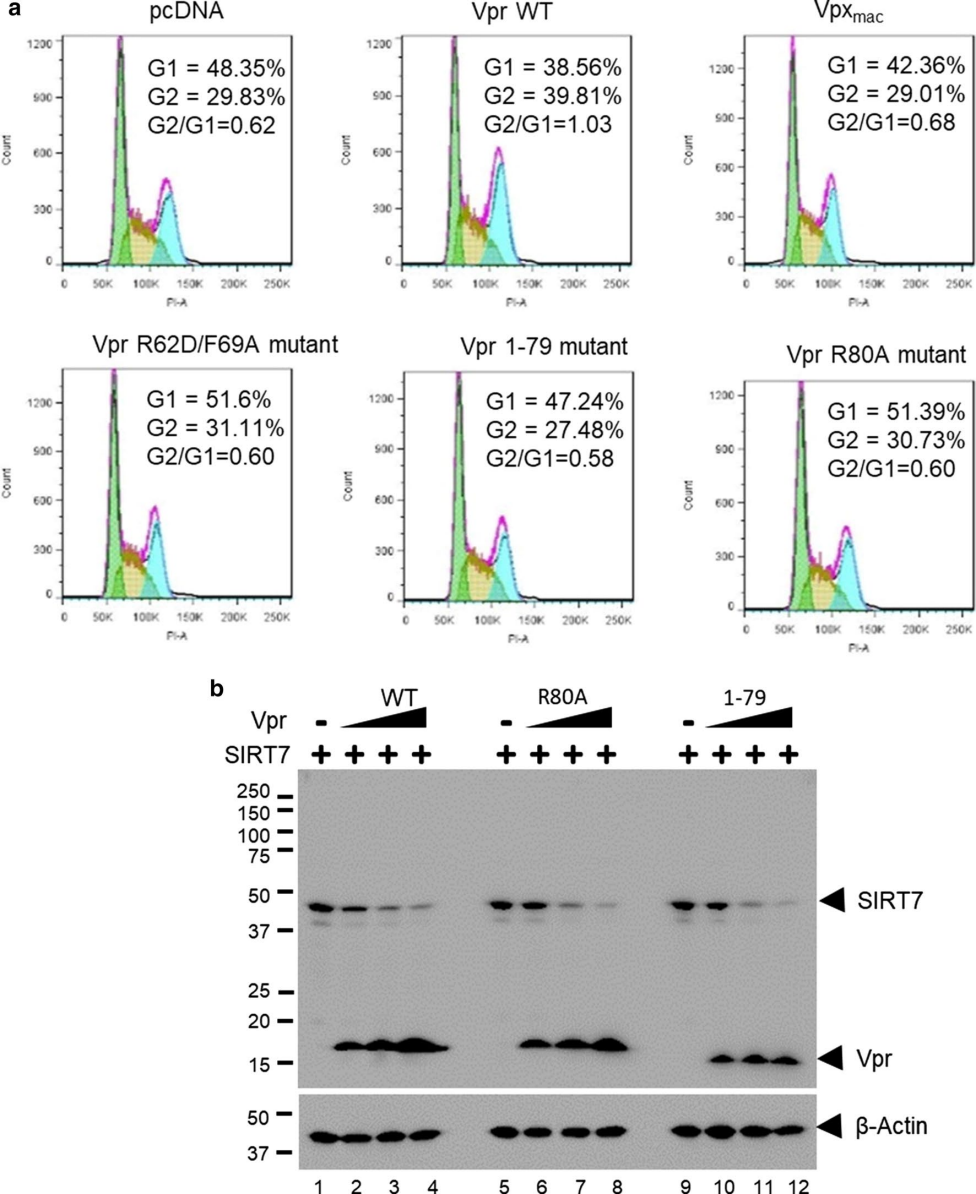
SIRT7 degradation and G2/M arrest are independent functions of HIV-1 Vpr. **a** Cell cycle analysis of HEK293T cells transiently transfected with control plasmids or plasmids expressing HIV-1 Vpr WT, Vpr R80A, Vpr 1–79 (deletion of C-terminal residues 80–96), Vpr R62D/F69A or SIVmac Vpx. The percentage of bulk transfected cells in G1or G2 and the ratio of G2/G1 are indicated. **b** HEK293T cells were co-transfected with plasmids expressing SIRT7 or Vpr WT, R80A or 1–79 mutant. Cell lysate was analyzed with immunoblotting after 48 h. The experiments were repeted two times with similar results

One distinctive phenotype that has been associated with HIV-1 Vpr expression in cycling cells is G2 arrest. HEK293T cells transfected with HIV-1 Vpr, but not with SIVmac Vpx, display accumulation in G2 (Fig. 5a, upper panels). Mechanistically, the C-terminus of Vpr has been proposed to recruit unidentified cell cycle related factor(s) onto the CRL4-DCAF1 for proteasome-dependent degradation, activating damaged DNA response pathways. Cell cycle analyses of HEK293T cells transfected with the Vpr mutant that is deficient for DCAF1-binding (R62D/F69A) or Vpr C-terminal mutants (residue 1–79, and R80A) confirmed this model (Fig. 5a, lower panels). Since SIRT7 participates in the damaged DNA response pathway, we tested whether the C-terminus of Vpr interacts with SIRT7 for its degradation (Fig. 5b). Both Vpr C-terminal mutants efficiently mediated SIRT7 degradation. Thus, Vpr-dependent SIRT7 degradation is independent of Vpr-induced G2 arrest.

## Discussion

**Fig. 6:**
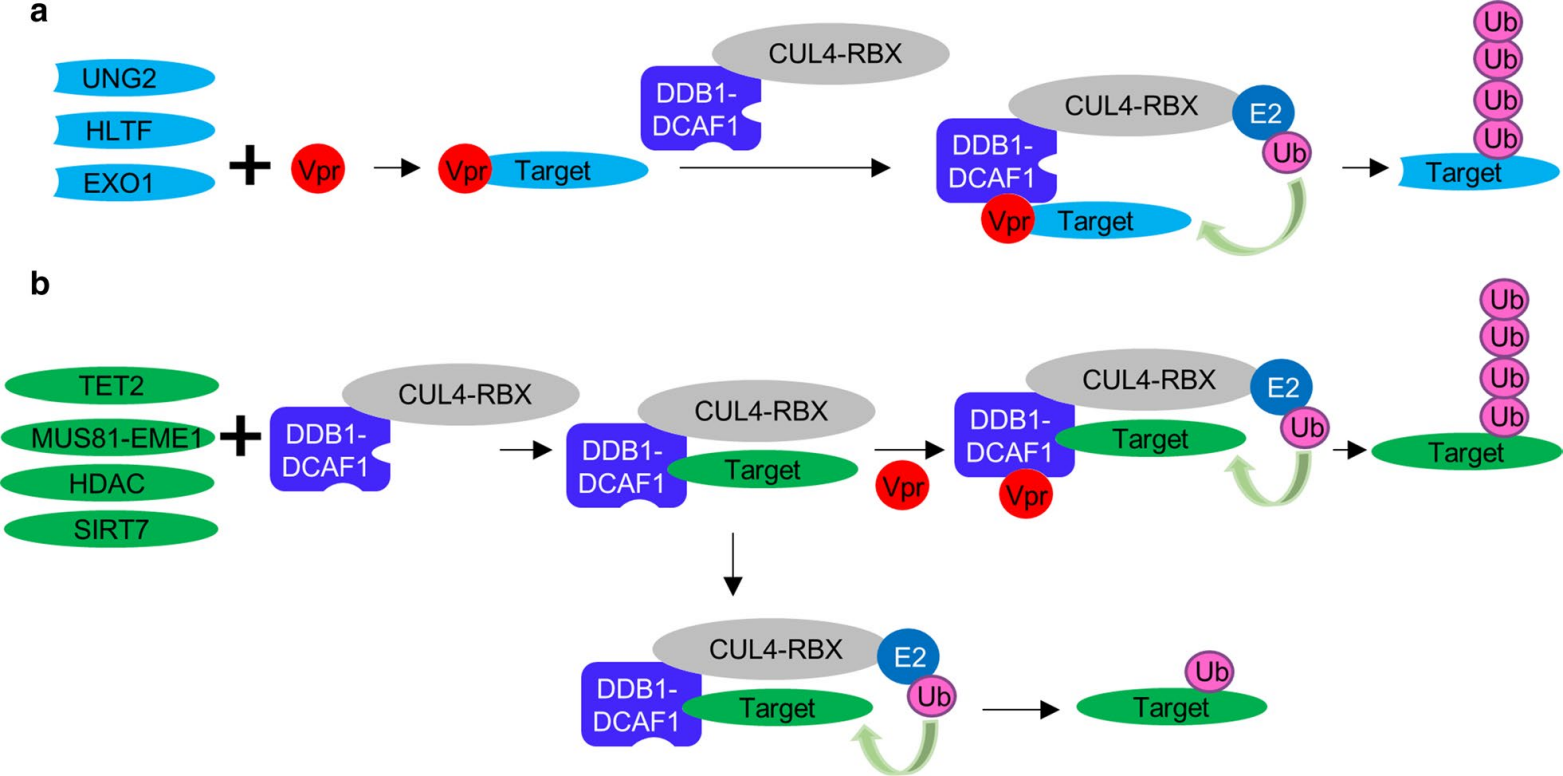
Two alternative modes of Vpr-mediated degradation of cellular targets via CRL4-DCAF1. **a** Vpr directly binds substrates and loads them onto the CRL4-DCAF1 for poly-ubiquitination. **b** The substrates are recruited to the CRL4-DCAF1 and some are mono-ubiquitinated. Vpr binds to the CRL4-DCAF1-substrate complex and promotes poly-ubiqiitination of the substrates. Vpr may or may not directly interact with the substrates

The majority of phenotypic effects exerted by HIV-1 Vpr are closely associated with its interaction with the CRL4-DCAF1 complex. Mechanistically, Vpr possesses two protein–protein interfaces, one of which interacts with DCAF1 while the other directly binds cellular targets and recruits them for poly-ubiquitination by the CRL4-DCAF1 complex, resulting in proteasome-dependent degradation (Fig. 6a). For example, Uracil DNA glycosylase 2 (UNG2), Helicase-like transcription factor (HLTF) and Exonuclease I (EXO1) directly bind to Vpr, which binds to DCAF1 and thus bridging the target to the CRL4 [15, 16, 27]. In addition to this canonical mechanism of action, which is also utilized by other viral proteins to hijack CRLs to overcome anti-viral responses, Vpr induces degradation of native CRL4-DCAF1 interacting proteins (Fig. 6b), such as MUS81-EME, HDAC and TET2 [5–7]. In this study, we show that Vpr mediates degradation of another CRL4-DCAF1 interacting protein, SIRT7. We find that ectopic expression of HIV-1 Vpr reduces cellular SIRT7 level, which can be rescued by CRL E3 ligase inhibitor MNL4924 and proteasome inhibitor MG132. In vitro reconstitution assays show that Vpr induces poly-ubiquitination of SIRT7 by the CRL4-DCAF1. Vpr proteins isolated from different HIV-1 lineages or from ancestral SIVs that infect chimpanzee, but not from HIV-2, show an ability to degrade SIRT7 in the same manner, suggesting that SIRT7 down-regulation is beneficial to HIV-1 infection.

Several studies have suggested a functional link between histone acetylation state and HIV-1 replication. Proviral cDNA, which is reverse transcribed from viral RNA upon entry into the cell, is integrated into the host genome; active transcription from the integrated genome has been correlated with post-translational modification of histones such as hyper-acetylation [28, 29]. Further, treatment of HIV-1 latently infected cells with histone deacetylase (HDAC) inhibitors results in the transcriptional activation of the HIV-1 promoter and an increase in HIV-1 replication [30]. Interestingly, HIV-1 Vpr targets class I HDACs on chromatin for proteasome-dependent degradation, by usurping the CRL4-DCAF1, and induces reactivation of latent HIV-1 provirus [6, 31]. In another pathway, Vpr-bound CRL4-DCAF1 degrades TET2 thereby inhibiting HDAC recruitment to the promoter of pro-inflammatory cytokine interleukin-6 (IL-6), whose expression enhances HIV-1 replication [7, 32]. Given that SIRT7 mediates transcriptional repression via histone 3 Lys18 acetylation [33], it is tempting to speculate that SIRT7 down-regulation by Vpr facilitates efficient HIV-1 replication. This possibility will be addressed in future studies.

Previous studies suggested that HIV-1 Vpr antagonizes several post-replication DNA repair machineries by usurping the CRL4-DCAF1. Importantly, recent reports showed that removal of HLTF and EXO1 is beneficial to HIV-1 replication [27, 34]. The observation that removal of these proteins only partially accounts for the phenotypic effect of Vpr implicates the existence of additional Vpr-counteracted machineries that restrict viral replication. Interestingly, Vpr recruits CRL4-DCAF1 to nuclear foci, where multiple DNA repair proteins, including SIRT7, are localized [10, 35]. The C-terminus of Vpr is essential for localization to nuclear foci as well as for induction of G2 cell cycle arrest, implying that degradation of nuclear foci bound cellular proteins induces DNA damage, leading to activation of the DNA damage response pathway and G2 arrest [35]. Since Vpr-mediated degradation of HLTF, EXO1 and SIRT7 is independent of Vpr-induced G2 cell cycle arrest, searches for additional Vpr targets are needed.

## Conclusions

HIV-1 Vpr mainly exerts its biological functions by interacting with the CRL4-DCAF1, thereby inducing proteasomal degradation of multiple host factors. We provide biochemical evidence that SIRT7 is another celluar target of Vpr from HIV-1 lienage.

## Data Availability

All data generated or anlalyzed during this study are included in the published article and all materials are available from the corresponding author on reasonable request.

## Abbreviations

CRL4: Cullin 4 RING E3 ubiquitin ligase
DDR: DNA damage repair
EXO1: Exonuclease I
HIV-1: Human immunodeficiency virus 1
HIV-2: Human immunodeficiency virus 2
HLTF: Helicase-like transcription factor
SIV: Simian immunodeficiency virus
Vpr: Viral protein R
UNG2: Uracil DNA glycosylase 2
SIRT7: Sirtuin 7
